# Intestinal Na^+^, K^+^, 2Cl^−^ cotransporter 2 plays a crucial role in hyperosmotic transitions of a euryhaline teleost

**DOI:** 10.14814/phy2.13028

**Published:** 2016-11-23

**Authors:** Andrew J. Esbaugh, Brett Cutler

**Affiliations:** ^1^ University of Texas at AustinMarine Science InstituteAustinTexas

**Keywords:** Euryhaline, ion regulation, NKCC, red drum, salinity transfer, V‐type ATPase, water absorption

## Abstract

Euryhaline fishes, such as the red drum *(Sciaenops ocellatus*), must quickly transition between hyperosmotic and hypoosmotic physiological strategies. When freshwater individuals transition to seawater they are exposed to increased diffusive water loss and ion gain. To maintain osmoregulatory balance these animals must drink and absorb seawater through the intestine, followed by ion excretion at the gills. The ability of fishes to transition between strategies can limit the magnitude of osmotic shock that can be tolerated. Here, we demonstrate that red drum can tolerate direct transfer from freshwater to full‐strength seawater with marginal impacts on osmotic balance, as indicated by plasma and muscle ion concentration, as well as muscle water. Seawater transition is concurrent with a significant increase in intestinal fluid volume. Typical patterns of osmoregulatory plasticity were observed in the gill with increased expression of *nkcc1* and *cftr*. Expression changes in the anterior intestine were observed after 24 h for *nkcc2* with smaller and later responses observed for *slc26a3, slc26a6,* and *nbc*. Immunofluorescence staining demonstrated similar patterns of NKCC localization in freshwater and seawater intestines; however, reduced basolateral staining of V‐type ATPase was observed in seawater. Electrophysiological preparations demonstrated that seawater fish had increased absorptive current in the anterior intestine, which was significantly reduced in the presence of 10 *μ*mol/L bumetanide. Overall, these results suggest that *nkcc2* plays a crucial role during hyperosmotic transitions, and may be a more important complement to the well‐known bicarbonate secretion pathway than generally considered.

## Introduction

Fishes are osmoregulating organisms and therefore must regulate their internal osmotic pressure in the face of variable external conditions. In freshwater, fishes constantly lose ions to the hypoosmotic environment while gaining water. To compensate, the gills of freshwater fishes contain specialized ionocytes capable of absorbing ions from the environment. The routes for Na^+^ uptake include apically oriented Na^+^ H^+^ exchangers (*nhe*) (Ivanis et al. 2008; Kumai et al. 2011), which sometimes work in concert with Rh proteins (Kumai and Perry 2011; Sinha et al. 2013), Na^+^ Cl^−^ cotransporters (*ncc*) (Hsu et al. 2014; Lin et al. 2015), as well as acid‐secreting ion channels (*asic*) (Dymowska et al. 2014, 2015). In addition to *ncc*, Cl^−^ uptake can also occur through a series of slc26 anion exchangers, most notably *slc26a3*,* slc26a4,* and *slc26a6* (Bayaa et al. 2009; Perry et al. 2009). The plethora of different osmoregulatory pathways led to the identification of distinct subpopulations of branchial ionocytes (Perry and Gilmour 2006; Hwang and Lee 2007; Hwang et al. 2011), defined by their specific transport mechanisms.

Marine teleosts must actively expel excess Na^+^ and Cl^−^ across their gills, which occurs via specialized ionocytes that contain three crucial proteins (see reviews Marshall 2002; Marshall and Grosell 2006). Basolateral Na^+^ K^+^ ATPase (*nka*) maintains both a low intracellular Na^+^ concentration and an intracellular‐negative transmembrane potential. The low intracellular Na^+^ establishes the electrochemical gradient required to concentrate Cl^−^ inside these cells through *nkcc1*. The internal negative potential and high Cl^−^ concentration allows for apical Cl^−^ excretion through *cftr*, which establishes an outside negative transepithelial potential that drives paracellular Na^+^ excretion. To counteract water loss to the hyperosmotic environment, marine teleosts must also drink and absorb seawater (see review Grosell et al. 2011). Esophageal desalination is driven in part by apical NHE (Hirano and Mayer‐Gostan 1976; Parmelee and Renfro 1983; Esbaugh and Grosell 2014) and makes the imbibed fluid isosmotic with the plasma as it enters the intestine, after which any further ion absorption will drive water absorption. A number of different proteins have been implicated in intestinal ion absorption, including *nkcc2*,* ncc*,* slc26a3, slc26a6*,* nbc* (*slc4a4*), and various *nhe* isoforms (Frizzell et al. 1979; Field et al. 1980; Grosell et al. 2007; Cutler and Cramb 2008; Taylor and Grosell 2008; Genz et al. 2011; Esbaugh et al. 2014). Their functions can also be modified through the action of ancillary proteins such as the V‐type H^+^ ATPase (Guffey et al. 2011), intestinal carbonic anhydrase (Sattin et al. 2010), and soluble adenylyl cyclase (Tresguerres et al. 2010). Despite the wide array of proteins, intestinal ion absorption can be divided into two pathways: bicarbonate dependent and independent. The former describes the anion exchange pathways that result in bicarbonate secretion and subsequent intestinal calcium carbonate precipitation (Wilson et al. 2002; Grosell 2006). The latter pathway refers to the electroneutral cotransport of Na^+^ and Cl^−^ via NKCC or NCC.

It is clear that osmoregulation in freshwater and seawater is very different, yet euryhaline species are capable of surviving in both environments. The red drum is one such species; tolerating direct transfer from full‐strength seawater to freshwater (Watson et al. 2014). This transition resulted in a modest, but persistent, decrease in plasma osmolality with no effect on muscle water content. Red drum accomplish this transition in part through downregulation of the branchial salt excretion mechanisms and reduced drinking, both of which occur within 24 h of transfer (Watson et al. 2014). Although not previously examined, this may also lead to a degree of atrophy whereby the mechanisms related to intestinal ion absorption are reduced. Because drinking seawater is paramount to marine osmoregulation, the ability of the intestine to regain full capacity for ion absorption is critical during return to hyperosmotic environments. In fact, recruitment of the gastrointestinal tract for osmoregulation makes transfer to seawater a distinct physiological challenge from freshwater transfer. Also of note is that red drum are a federally protected, economically important sportfish species, which further highlights the importance of ascertaining the underlying physiological mechanisms that allow them to tolerate environmental change.

On this background, this study sought to examine the effect of acute seawater transfer on freshwater‐acclimated red drum. We first examined the effect of transfer on water and ion balance in the plasma and muscle over the 7 day posttransfer window. The intestinal fluid contents were monitored over this time frame as an indirect measure of the initiation of drinking and water absorption. Intestine and gill samples were collected to assess gene expression of nine target osmoregulatory genes. Immunofluorescence and electrophysiological Ussing chamber preparation were then used to further assess the osmoregulatory plasticity of the intestine and validate gene expression data at a functional level.

## Material and Methods

### Animals and experimental design

Animal experiments were performed with the approval of the University of Texas at Austin Institutional Animal Care and Use Committee. All fish were raised from the fry to juvenile stage on site at the Fisheries and Mariculture Lab at the University of Texas Marine Science Institute (UTMSI). Fry were either raised from embryos on site or provided by the Texas Parks and Wildlife Hatchery located in Corpus Christi, Texas, USA. All fish are raised on commercial fish feed in recirculation systems maintained at 22°C with constant aeration. To develop a freshwater phenotype, juvenile fish (45 ± 2 g; *N *=* *30) were transferred directly to an approximately 1000‐L circular holding tank equipped with biofiltration and aeration containing dechlorinated Port Aransas tap water (mM; Na^+^ 6.7, Cl^−^ 5.8, Ca^2+^ 9.1, Mg^2+^ 0.9, K^+^ 0.3, titratable alkalinity 2.0, pH 8.1). Fish were maintained in freshwater for at least 14 days prior to experimentation. Note that previous work in our laboratory showed that the physiological events that accompany freshwater transfer are complete by 24 h posttransfer (Watson et al. 2014), and therefore 14 weeks was deemed suitable. Three identical tanks containing full‐strength seawater (35 ppt) were used for acute seawater transfer. Seawater was obtained from the channel directly adjacent to UTMSI, and was subject to settlement, sand filter filtration, and UV sterilization. The transfer procedure consisted of manually netting fish from the freshwater tank and transferring to one of three seawater tanks (*N *=* *8 each) – one for each time point –, whereas the freshwater fish were netted but returned to the freshwater tank. All fish were starved for 24 h prior to transfer and 48 h prior to sampling to ensure that no food was present in the intestine. Freshwater fish were sampled 24 h after pseudotransfer and seawater fish were sampled after 24 h, 72 h, and 7 days. For sampling, fish were anesthetized in MS‐222 (250 mg/l buffered with 500 mg/L NaHCO_3_) followed by spinal transection. Blood was collected by caudal puncture and plasma was isolated by 1 min centrifugation at 2000 g. The intestine was exposed by a ventral incision using a scalpel and scissors after which the connective tissue was gently removed and the intestine elongated. Hemostats were used to clip the anterior intestine immediately after the pyloric cecae and the posterior intestine just prior to the rectum. The intestine was then excised and the fluid was drained into a preweighed microcentrifuge tube. Muscle tissue was excised from the dorsal side of the fish. All gill arches were excised, lamellar tissue was separated, and the sample was frozen immediately on dry ice. The anterior intestine (defined as the first third of the excised portion) was subsampled for histology samples, which were placed immediately in the zinc formalin Z‐Fix fixative (Anatech). The mucosal epithelia of the remaining portion was sampled for RNA analysis by scraping the epithelium with a glass slide, with the resulting sample frozen immediately on dry ice.

A second series of experiments was performed to obtain samples for in situ electrophysiology procedures. The experimental design is as described above with the exception that seawater fish were transferred to either freshwater or seawater tanks and allowed to acclimate for at least 7 days prior to experimentation. As above all fish were starved for at least 48 h prior to sample collection and anterior intestine samples were collected as described above.

### Gene expression

Total RNA was extracted using TRI Reagent (Molecular Research Center Inc, Cincinnati, OH) according to manufacturer guidelines; homogenization was performed using motor‐driven tissue homogenizer. Total RNA was quantified using an ND‐1000 (Thermo Scientific, Waltham, MA) spectrophotometer at a wavelength of 260 nm and sample purity was assessed using 260:280 ratios. A 1 *μ*g sample of RNA was DNase treated with amplification grade DNase I (Thermo Scientific; manufacturer specifications) to remove potential DNA contamination. The resulting DNase‐treated RNA was reverse transcribed using RevertAid (Thermo Scientific), according to manufacturer specifications. Real‐time PCR was performed on an Mx3000P real‐time PCR system (Stratagene, Santa Clara, CA) using the Maxima SYBR green master mix kit (Thermo Scientific; 12.5 *μ*L reactions). Primer sets were designed using the Primer3 freeware (Untergasser et al. 2007, 2012) based on sequence information from an in‐house intestine, gill, and red blood cell transcriptome. Specificity of each set was verified using standard PCR and gel electrophoresis. The thermocycler program and reaction composition were performed according to manufacturer guidelines at an annealing temperature of 58°C, and dissociation curves were used to assess the primer specificity of each reaction. The PCR efficiency of each primer pair was calculated using a cDNA standard curve. PCR efficiencies ranged from 90 to 113% with an *R* ^2 ^≥ 0.98. Relative mRNA expression was calculated using the delta–delta ct method using elongation factor 1α (*ef1α*) as an internal control (Tang et al. 2007) and the seawater treatment as the relative control (Pfaffl 2001). Successful DNase treatment was verified using a no reverse transcriptase control for each tissue set. Primer sequences were described previously as follows: *ef1α*,* nbc, nkcc1, nkcc2, CA‐c* (Esbaugh et al. 2016). Additional primer sequences can be found in Table 1.

**Table 1:** Real‐time PCR primers used for gene expression analysis. See methods for citations of primers not listed here

Gene	Accession #	Orientation	Sequence
*VHA,* β *subunit*	KU899108	F	CCT ACC ATT GAG CGT ATC ATC A
		R	CGT AGG AGC TCA TGT CAG TCA G
*slc26a3a*	KX507131	F	CAA CTG TTG CTT CTT TGA CGA C
		R	AGT CTG GGT GTT TCT CGA TGA T
*slc26a3b*	KX507132	F	CAA GGC ACA CAG GAA AAT CA
		R	GGG AAG CTC TTC CAT GTT CA
*slc26a6*	KX507133	F	TGC CAG GGA TTT TGA TCT TC
		R	GTT TCT TCT TTG CCG ACA GG

**Table 2:** The effects of seawater transfer on additional plasma, muscle, and intestinal osmoregulatory parameters in freshwater‐acclimated red drum, *S. ocellatus*

		Freshwater	24 h post	72 h post	7 days post
Muscle					
Water (%)		78.9 ± 0.2	78.9 ± 0.2	78.1 ± 0.8	78.1 ± 0.3
Na^+^ (mmol kg^−1^)		29 ± 2	29 ± 1	28 ± 1	28 ± 2
Cl^−^ (mmol kg^−1^)		32 ± 4	33 ± 3	30 ± 2	27 ± 3
Ca^2+^ (mmol kg^−1^)		1.7 ± 0.2	1.9 ± 0.1	2.0 ± 0.1	1.7 ± 0.2
Mg^2+^ (mmol kg^−1^)		21 ± 2	22 ± 1	23 ± 1	21 ± 2
Plasma					
Ca^2+^ (mmol/L)		1.2 ± 0.1	1.1 ± 0.1	1.2 ± 0.1	1.2 ± 0.1
Mg^2+^ (mmol/L)		1.1 ± 0.1	1.4 ± 0.1	1.1 ± 0.1	1.4 ± 0.1
Intestinal Fluid					
Na^+^ (mmol/L)		—	76 ± 14	47 ± 4	39 ± 8
Total CO_2_ (mmol/L)		—	39 ± 4	32 ± 7	32 ± 12
Pellet (mg kg^−1^)		—	160 ± 113	5 ± 2	21 ± 11

No significant differences were detected for any variable (ANOVA; *P *>* *0.05, or ANOVA on Ranks; *P > *0.05). *N*: muscle and plasma = 6–8; intestinal fluid: pellet = 5–7, total CO_2_ = 3–7, Na^+^ = 4–7.

### Immunohistochemical analysis

All samples were fixed in Z‐Fix zinc formalin fixative (Anatech) for 24 h at room temperature. Following fixation, tissue was dehydrated in 95% ethanol for three cycles of 1 h followed by three cycles of 45 min in 100% ethanol. Tissue samples were then treated twice with Histochoice clearing reagent (Amresco, Solon, OH) for 90 min, followed by two 60 min washes in Paraplast Plus embedding media (Leica Biosystems Richmond Inc., Buffalo Grove, IL) at 58°C. The samples were embedded in casting cubes at room temperature and stored at 4°C until sectioning. Samples were sectioned at 10 *μ*m using a microtome and mounted on Superfrost Plus slides (VWR, Houston). Each slide contained at least two sections so a no‐primary antibody controls could be run concurrent with each sample. Each slide was deparaffinized and rehydrated as follows: two washes of 5 min in Histochoice clearing agent, two washes of 5 min in 100% ethanol, a 5 min wash in 95%, and a final wash for 5 min in 70% ethanol. An antigen recovery protocol was performed, which consisted of immersing the slides in 10 mmol/L citrate buffer (pH 6.0; 0.05% Tween 20) and heating in the microwave for three cycles of 5 min. Individual sections were isolated using a hydrophobic barrier marker and the sections were immersed in 1% SDS in PBS for 5 min. Tissues were treated with blocking solution (0.5% Triton X, 1% fetal calf serum in PBS) twice for 5 min, followed by primary antibody incubation for either 90 min (NKCC, NKA) at room temperature or overnight (VHA, NKA) at 4°C. Note that immunofluorescence was limited to these three proteins owing to the limited availability of commercial antibodies to nonmodel fish species. Samples were rinsed with blocking solution three times for 5 min followed by a 1 h incubation with secondary antibody (1:500 dilution in blocking solution). Samples were then stained with Sudan Black for 10 min – to reduce background fluorescence – rinsed twice with blocking buffer and mounted with vector shield mounting media with DAPI. A primary monoclonal antibody for NKCC (t4), originally developed by Lytle/Fosbush, was obtained from the Developmental Studies Hybridoma Bank (DSHB) developed under the auspices of the NICHD and maintained by The University of Iowa, Department of Biology, Iowa City, IA 52242. This antibody has been previously verified for fishes as indicated by the manufacturer, and also previously used on red drum (Watson et al. 2014). A polyclonal NKA antibody developed in rabbit was obtained commercially (catalog # sc‐28800; Santa Cruz Biotechnology, Dallas, TX), which has also been previously shown to be effective in fishes (Ruhr et al. 2014). Also note that this antibody gives identical staining patterns to the well‐known α5 mouse monoclonal NKA antibody available through DSHB. The VHA antibody (mouse monoclonal) was obtained commercially and targets the β1/2 subunits (catalog # sc‐271832; Santa Cruz Biotechnology). The target epitope was first assessed by aligning the red drum sequences with known successful targets (human and mouse), and specificity was assessed using Western blot (see below). This resulted in a single band of approximately 55 kDa, which corresponds to the published molecular weight.

### Western blot

Protein was isolated from intestine and gill tissue using RIPA buffer (Amresco), according to manufacturer protocols. RIPA buffer was supplemented with Protease Inhibitor Cocktail (Amresco) according to manufacturer guidelines. Tissue (20 mg/mL RIPA) was homogenized by sonication at 180 W for 2 min using 10 sec on/off cycles. The homogenate was centrifuged at 14,000*g* for 15 min at 4°C and the resulting supernatant was removed. Protein was quantified using the bicinchoninic acid assay (G Biosciences, St. Louis, MO) according to manufacturer guidelines and using commercial albumin standards (Thermo Scientific). Protein was aliquoted and stored at −80°C prior to use. SDS‐PAGE was performed using a Bio‐Rad mini‐protein electrophoresis system. Protein of 25 *μ*g (20 *μ*L) was loaded onto precast 4–15% Mini‐PROTEAN TGX stain‐free gels (Bio‐Rad). Separation was performed at 250 mV for approximately 1 h and transfer of protein to a PVDF membrane was performed using a Trans‐Blot Turbo transfer system (Bio‐Rad), according to manufacturer guidelines. Transfer success was evaluated with the stain‐free detection settings using a Chemi‐doc MP imaging system (Bio‐Rad). The blot was then rinsed in PBST and blocked for 1 h in PBST with 5% skim milk. The blot was incubated with primary VHA antibody at 1:500 dilution in blocking buffer at 4°C overnight. The blot was then washed in PBST and incubated with HRP‐conjugated anti‐rabbit secondary antibody (1:10,000; Thermo Scientific) for 1 h, after which the blot was washed in PBST. Visualization was performed using the Clarity Western ECL Substrate (Bio‐Rad) and a Chemi‐doc MP imaging system using autodetect chemiluminescence settings.

### Analytical methods

For muscle water and ion content analysis the tissue was weighed and placed in a dehydrating oven at 55–60°C. Samples were weighed daily until a stable value was reached. The difference in initial wet weight and dry weight was calculated as water content. Dried samples were subsequently dissolved overnight (55–60°C) in five volumes of 1 mol/L HNO_3_ acid. Samples were then vortexed and centrifuged for 3 min at 10,000*g* and the supernatant was collected and stored (4°C) for ion analysis. The tube containing intestinal fluid was weighed and centrifuged 15 min at 10,000*g* to pellet CaCO_3_ and any organic material. The fluid was removed by pipette and the tube was again weighed to obtain the intestinal fluid volume measures. The pellet was rinsed with deionized water and dried overnight at 45°C after which the pellet was digested with 1 mL of 5% sodium hypochlorite to remove organic material, as previously described (Al‐Jandal et al. 2011). The pellet was then again rinsed, dried, and weighed. All cation analysis was performed using an iCE 3000 atomic absorption spectrophotometer (Thermo Scientific). Chloride concentration was determined using a model 926 chloride analyzer (Cole Parmer). Fluid osmolality was determined using a Vapro 5520 osmometer (Wescor) with associated standards. Total CO_2_ was assessed using a model 965 carbon dioxide analyzer (Olympic Analytical Services).

### Electrophysiology

Electrophysiological recordings were performed on isolated anterior intestine epithelia as previously described (Esbaugh and Grosell 2014; Ruhr et al. 2014). Briefly, the anterior intestine of fasted animals (48 h) was dissected from red drum, immediately mounted in a 0.4 cm^2^ surface area tissue holder, and placed between two Ussing half chambers (Physiologic Instruments). Each chamber contained 2 mL of serosal style saline: 151 mmol/L NaCl, 3 mmol/L KCl, 0.88 mmol/L MgSO_4_, 5 mmol/L NaHCO_3_, 1 mmol/L CaCl_2_, 0.5 mmol/L NaH_2_PO_4_, 0.5 mmol/L KH_2_PO_4_, 4.5 mmol/L urea, 5 mmol/L glucose, 3 mmol/L HEPES (Na^+^ salt), and 3 mmol/L HEPES (free acid). Temperature was maintained at 25°C by a recirculating water bath and both chambers were aerated by a 0.3% CO_2_ in oxygen mixture, as controlled using a pair of gas mass controllers (Sierra Instruments and Cole Parmer). Saline was aerated for at least 60 min prior to experimentation. Short‐circuit current (*I*_sc_) was measured under symmetrical voltage clamp conditions using current and voltage electrodes (Physiologic Instruments; P2020‐S) connected to amplifiers (Physiologic Instruments; VCC600). Conductance of the epithelium was determined by passing 3 sec pulses of 1 mV from mucosal to serosal bath every 60 sec. To inhibit NKCC activity a final concentration of 10 *μ*mol/L bumetanide, or 0.1% DMSO vehicle control, was added to the mucosal saline bath after stable control values had been obtained. In all cases the effects of bumetanide were apparent in minutes, but tissues were allowed to stabilize for final determinations. In all cases, reported data represent the average of a stabilized tissue over 10 min.

### Statistics

Statistical comparisons across time points were performed using an analysis of variance (ANOVA) for all ion and water content data, as well as gene expression data. Intestinal magnesium concentration was compared using a student's *t*‐test. All gene expression data were log transformed prior to statistical analyses and normality and equal variance was confirmed prior to analysis. In cases where data were not normally distributed or had unequal variance an ANOVA on ranks was performed. All ANOVA analyses were followed by a Holm–Sidak post ‐hoc test versus the control group and ANOVA on ranks analyses were followed by a Dunn's post hoc test versus control. Electrophysiology data were assessed using a two‐way ANOVA. All analyses were performed using SigmaPlot 12.5 and a *P* ≤ 0.05.

## Results

### Fluid and muscle osmolytes

**Figure 1:**
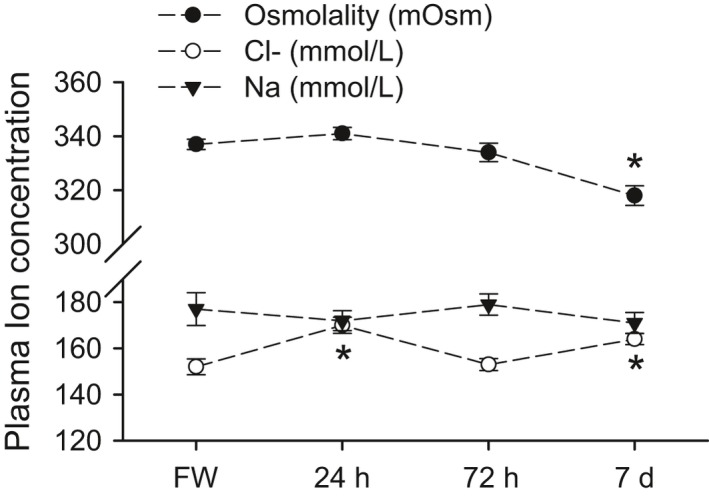
Plasma osmolality, sodium, and chloride in red drum acclimated to freshwater and acutely transferred to full‐strength seawater. Asterisks denote significant differences from the freshwater control time point (One‐way ANOVA; *P *≤* *0.05; *N *=* *5–8). All values are mean ± S.E.M.

**Figure 2:**
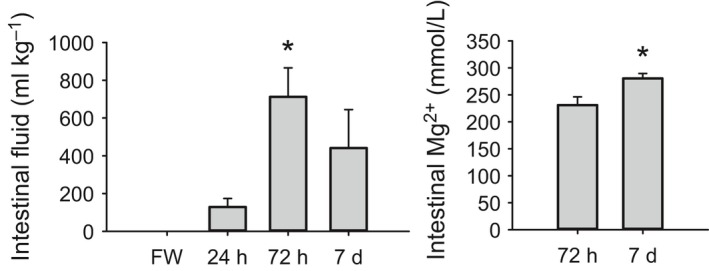
Intestinal fluid volume (A) and fluid magnesium concentration (B) in freshwater‐acclimated red drum acutely transferred to full‐strength seawater. (A) Asterisk denotes significant difference from 24 h time point (ANOVA on ranks; *P *≤* *0.05; *N *=* *7–8). (B) Asterisk denotes a significant difference between time points (*t*‐test; *P *≤* *0.05; *N *=* *7–8). All values are mean ± S.E.M.

Transfer of red drum from freshwater to full‐strength seawater resulted in a significant decrease in plasma osmolality by 7 days posttransfer; intermediate time points were not significantly different from control (Fig. 1; one‐way ANOVA). Only plasma chloride was significantly affected by seawater transfer with significant, but transient, increases at 24 h and 7 days posttransfer (Fig. 1 and Table 2). Seawater transfer had no effect on muscle water balance at any time point, nor were there any differences in observed muscle ion concentrations at any time point (Table 2). No intestinal fluid was obtained from freshwater fish, whereas 20 of 24 seawater‐acclimated individuals had intestinal fluid. When normalized to body mass this trend was only statistically significant at 72 h (Fig. 2a). Of these fluid samples, only a subset had sufficient volumes for ion chemistry. When assessing between the three posttransfer time points, no differences were observed in fluid Na^+^ or total CO_2_ concentrations. Sufficient sample volume for measuring Mg^2+^ was only obtained from nine individuals from the 72 h and 7‐day posttransfer time points. A significantly higher Mg^2+^ concentration was observed at 7 days posttransfer (student's *t*‐test, *P *=* *0.03). Of the 20 individuals with intestinal fluid, only 12 had quantifiable CaCO_3_ precipitates. There were no differences in precipitate amount between posttransfer time points and there was also no observable relationship between intestinal fluid volume and amount of precipitate (data not shown; linear regression, *P *=* *0.37, *R* ^2^ = 0.05).

### Gene expression and immunohistochemistry

**Figure 3:**
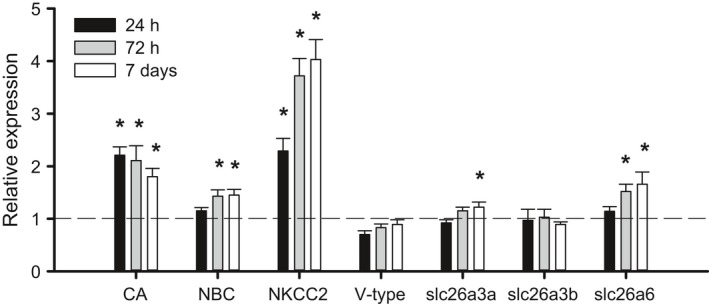
Relative mRNA expression of osmoregulatory genes in the anterior intestine of freshwater‐acclimated red drum after acute transfer to full‐strength seawater. All data are relative to the freshwater (FW) control and normalized using elongation factor 1*α*. Asterisks denote significant differences from freshwater control (One‐way ANOVA; *P *≤* *0.05; *N *=* *6–8). All values are mean ± S.E.M.

**Figure 4:**
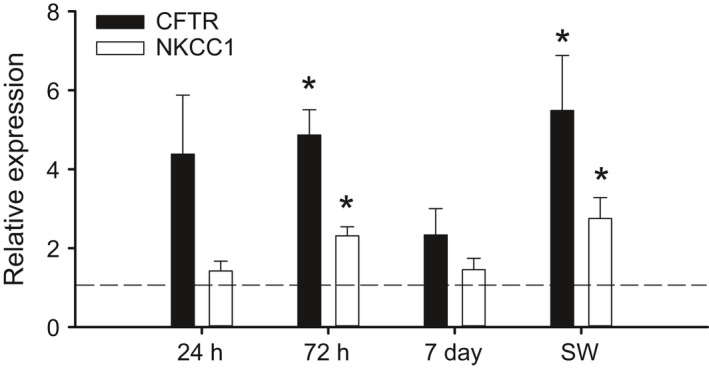
Relative mRNA expression of *cftr* and *nkcc1* in the gills of freshwater‐acclimated red drum after acute transfer to full‐strength seawater. All data are relative to the freshwater (FW) control and normalized using elongation factor 1*α*. Note the SW treatment represents a set of red drum never exposed to freshwater. Asterisks denote significant differences from freshwater control (One‐way ANOVA; *P *≤* *0.05; *N *=* *6–8). All values are mean ± S.E.M.

Seven genes relevant for intestinal water absorption were identified in our in‐house red drum transcriptome. Of these genes, six are related to the intestinal bicarbonate secretion mechanism, whereas *nkcc2* is bicarbonate independent. A Na^+^, Cl^−^ cotransporter (*ncc1*) was not identified in our transcriptome, whereas two isoforms of *slc26a3* were found. Seawater transfer caused significant upregulation in five of seven genes (Fig. 3A). *Nkcc2* and *CA‐c* showed the fastest response with significant upregulation by 24 h posttransfer. These genes also showed the largest increase in expression with a peak of 2.5‐ and 4‐fold for *CA‐c* and *nkcc2*, respectively. A significant increase of less than twofold was observed for *nbc*,* slc26a3a,* and *slc26a3b*. The branchial expression of *nkcc1* and *cftr* was also assessed as a consequence of seawater transfer. Both *nkcc1* and *cftr* showed significant upregulation after transfer, which occurred at 72 h posttransfer (Fig. 4). Neither 24‐h nor 7‐day time points were significantly different from freshwater controls. As a secondary point of comparison, we included a long‐term seawater gill sample from fish never transferred to freshwater. Expression values for both *nkcc1* and *cftr* were elevated above freshwater expression and were statistically similar to the 72 h posttransfer time point.

**Figure 5:**
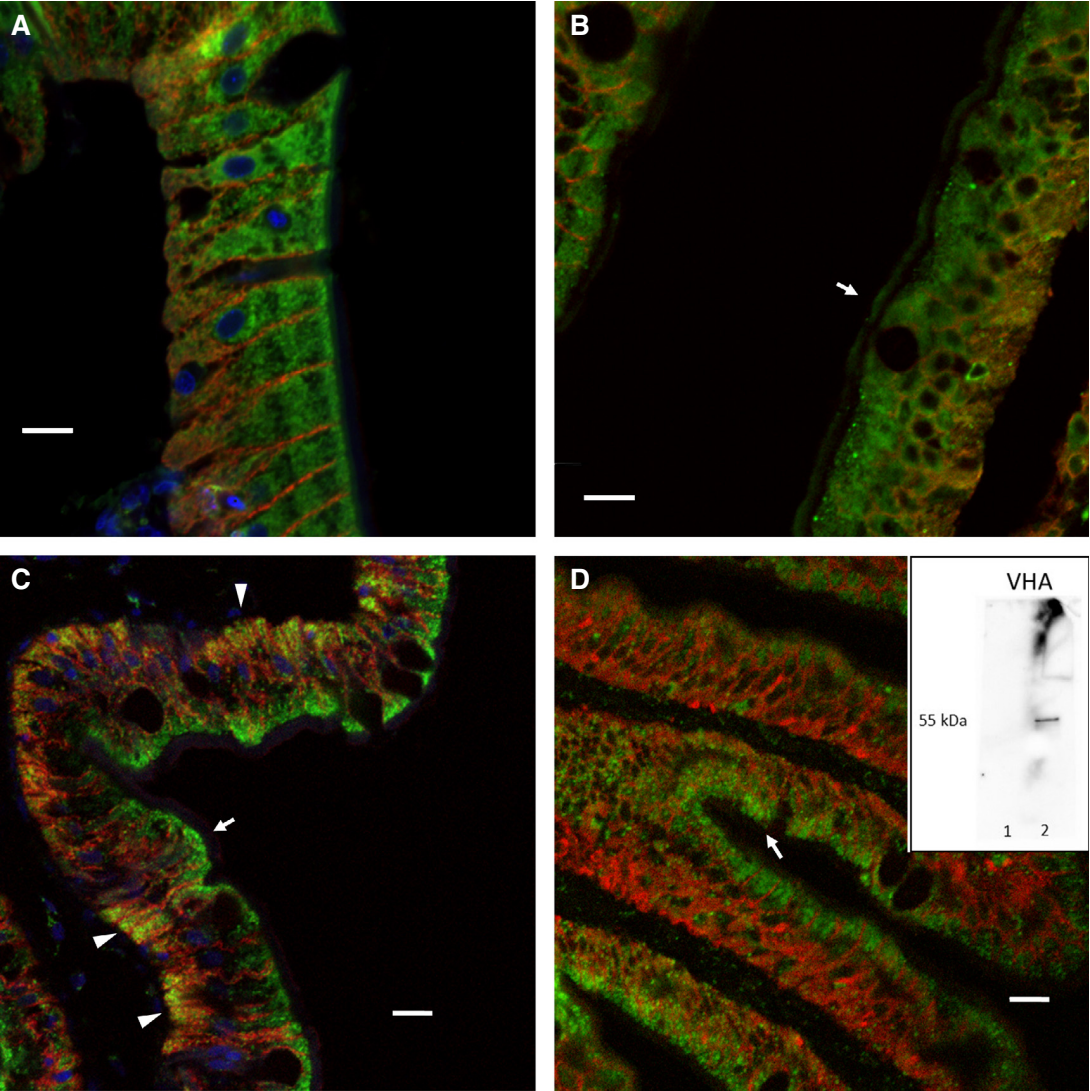
Representative immunofluorescence imaging of the anterior intestine of freshwater‐ (A, C) and seawater (B, D)‐acclimated red drum. (A, B) Colocalization of NKCC (green) and NKA (red). The arrow shows the apical type staining observed sporadically in the seawater intestine. (C, D) Colocalization of VHA (green) and NKA (red). The yellow shading denoted by the arrowheads shows basolateral colocalization in freshwater intestines, which was not observed in seawater‐acclimated fish. Subapical green shading in both freshwater and seawater fish is denoted by an arrow. Where present, blue shading (DAPI) represents cell nuclei. Scale bars equal 10 *μ*m. Inset: Western blot of isolate membrane and cytoplasmic protein fractions demonstrating VHA antibody specificity.

The tissue localization of V‐type H^+^ ATPase (VHA) and NKCC protein was assessed using immunofluorescence microcopy (Fig. 5; *N *=* *3). Note that NKCC antibodies are typically ineffective at differentiating NKCC1 and NKCC2. Nonetheless, clear apical NKCC staining was observed in the seawater‐acclimated fish, whereas no such staining was observed in freshwater‐acclimated fish (Fig. 5A, B). Different localization in seawater and freshwater was also observed for VHA. In freshwater, VHA was found in two distinct locations (Fig. 5C). Clear colocalization of VHA and NKA was observed demonstrating at least partial basolateral localization. Substantial subapical cytoplasmic staining was also observed in freshwater; however, clear apical membrane staining was not observed. No basolateral expression of VHA was observed in seawater‐acclimated fish (7 days posttransfer), whereas similar subapical cytoplasmic staining was observed in seawater. Substantial cytoplasmic and basolateral NKCC staining was observed in both freshwater and seawater. It is important to note that apical staining for both VHA and NKCC may be difficult to observe due to the substantial cytoplasmic signal, and even in the case of NKCC the observed apical staining was sporadic. Therefore, these findings should be interpreted with caution. Note that this study represents the first use of the VHA antibody in red drum, and as such Western blots were performed to verify specificity (Fig. 5D; Inset). As expected, only a 55 kDa band was observed, which was only associated with membrane protein fractions.

### Electrophysiology

**Figure 6:**
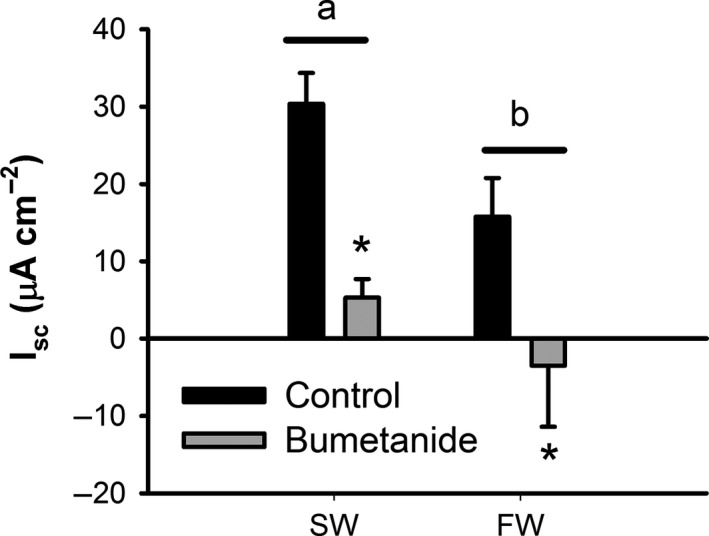
Short‐circuit current (*I*_sc_) data from the anterior intestine of the freshwater (FW)‐ and seawater (SW)‐acclimated red drum before and after incubation with 10 *μ*mol/L bumetanide. Positive values represent an absorptive ion current. Letters denote differences in *I*_sc_ between freshwater‐ and seawater‐acclimated fish, whereas asterisks denote a significant effect of bumetanide (Two‐way ANOVA; *P *≤* *0.05 (salinity); *P *≤* *0.05 (bumetanide); *N *=* *8–9). Note that there was not a significant interaction between treatments (*P *=* *0.37). All values are mean ± S.E.M.

The effect of gene expression changes on epithelial transport characteristics was assessed using a voltage clamp experimental design (Fig. 6). Seawater‐acclimated individuals had a more absorptive epithelia under symmetrical conditions, as evidenced by a significantly higher *I*_sc_ (two‐way ANOVA, see Fig. 6 caption for *P* values). Bumetanide was used to assess the contribution of NKCC2 to the absorptive current. A significant reduction in *I*_sc_ was observed in both freshwater‐ and seawater‐acclimated fish, with no statistically significant difference in the magnitude of inhibition. Bumetanide completely abolished the absorptive current in freshwater fish while eliminating 85% of the absorptive current in seawater fish. There was no effect of salinity or bumetanide treatment on epithelial conductance (data not shown; G = 12.7 ± 6.7, *N *=* *17).

## Discussion

Red drum are an estuarine‐dependent species that rely on these important coastal habitats as nursery grounds during larval and subadult life stages. While this species is typically associated with marine habitats – there are no known breeding freshwater populations – the seasonal and spatial variation in salinity within estuaries makes transitions between freshwater and seawater environments a likely physiological challenge. This study demonstrated that freshwater‐acclimated red drum were capable of direct transfer to full‐strength seawater without significant systemic osmoregulatory consequences. Direct transfer caused only a small and transient increase in plasma Cl^−^ with no effect on other ions. In fact, the overall effect of seawater transfer was a decrease in plasma osmolality after 7 days of acclimation. This counterintuitive finding is likely the result of a switch in osmoregulatory strategy from ion absorption to ion excretion. The ability of red drum to defend plasma osmolality was also extended to the tissues, as there was no change in muscle water or ion concentrations at any posttransfer time point. The defense of plasma osmolality, muscle water, and muscle ion concentrations during the initial transfer interval is atypical for freshwater to seawater challenges. The first 24 h of transfer is typically associated with the most dramatic changes (e.g., Bystriansky and Schulte 2011; Tipsmark et al. 2008); however, we have previously demonstrated similar patterns in red drum during freshwater transfer (Watson et al. 2014). This may suggest that red drum is a particularly adept species at such transitions, which would provide a clear physiological benefit during estuarine residence where salinities can fluctuate rapidly.

Acute seawater transfer in red drum is accompanied by a delayed drinking response, as assessed indirectly by intestinal fluid content. While intestinal fluid was observed in five of eight individuals sampled at 24 h, the peak fluid volume was not observed until 72 h posttransfer. As a point of comparison, acute transfer of killifish (*F. heteroclitus*) from isosmotic water to seawater resulted in a peak drinking response after only 12 h (Scott et al. 2008). Similarly, larval tilapia showed increased drinking rates within hours of a hyperosmotic challenge (Lin et al. 2001). This study only provides indirect evidence of drinking; however, these data still clearly demonstrate that the drinking response in red drum is more slowly initiated than that described for other species. Presumably, this delayed drinking response is directly related to the ability of red drum to resist plasma osmolality changes during hyperosmotic transition, which may also delay the blood pressure changes that can stimulate drinking reflex (see review Grosell et al. 2011). The degree of intestinal fluid processing can also be estimated using the fluid magnesium concentration (Genz et al. 2008). The intestinal fluid collected at 72 h posttransfer had magnesium concentrations representative of a 78.1 ± 1.5% fractionally absorbed sample, which increased to 82.1 ± 0.6% by 7 days posttransfer. These values are on the higher end of the fractional absorption range for seawater fishes, with estimates ranging from 38.5 to 84.9% (Wilson et al. 1996, 2002; Genz et al. 2008).

A qualitative assessment of the intestine was performed using immunofluorescence to specifically explore changes in protein localization in response to salinity acclimation. The general localization of NKCC protein was consistent between freshwater‐ and seawater‐acclimated fish. In both cases, the majority of immunofluorescence was localized to the cytoplasmic and subapical region. Some more obvious apical staining was observed in the intestines of the seawater‐acclimated fish; however, this was only sporadically observed. Unfortunately, the commercially available NKCC antibodies react to both NKCC1 and NKCC2, so these data must be interpreted with caution. In contrast, the localization of VHA was qualitatively different between freshwater and seawater‐acclimated fish. Most notably, there was clear basolateral localization in freshwater‐acclimated individuals, as evidenced by yellow coloration in Figure 5C. The basolateral VHA was absent in seawater‐acclimated fish with the majority of localization occurring in the subapical region. This generally supports previous work in marine fish intestines, which provided functional support for a role of apical VHA in facilitating apical HCO_3_^−^ secretion (Guffey et al. 2011). The basolateral localization in freshwater may suggest an increased tendency to transport protons back into the plasma during intracellular acid‐base disturbances.

The onset of drinking after seawater transfer coincided with a general increase in the physiological pathways involved in intestinal seawater processing. The earliest responses were observed for *nkcc2* and *CA‐c* expression, both of which were upregulated at 24 h posttransfer. The expression of *nkcc2* was further increased by 72 h posttransfer. Later upregulation was observed for *nbc, slc26a3a,* and *slc26a6*. It is also notable that the amount of total CO_2_ – an indicator of CO_3_^2−^/HCO_3_^−^ concentration – was approximately half that previously reported for seawater‐acclimated killifish, rainbow trout, and toadfish (McDonald and Grosell 2006; Genz and Grosell 2011; Genz et al. 2011). As such, the weight of evidence suggests that bicarbonate‐independent water absorption pathways may dominate in posttransition red drum, and are clearly more responsive to acute salinity transitions. The upregulation of *nkcc2* in response to salinity transfer was further supported at the functional level through exploration of *I*_*sc*_. Both freshwater and seawater intestines showed a significant decrease in *I*_*sc*_ in the presence of bumetanide, which is specific to NKCC‐type transporters. In fact, the magnitude of inhibition also supports the finding that NCC was not found in our red drum intestine and gill transcriptome. More importantly, the overall absorptive current in seawater‐acclimated intestinal epithelia was significantly greater than in freshwater. These data are indicative of increased absorption via active transport, presumably through increased basolateral NKA activity linked to apical NKCC2. However, *I*_*sc*_ is not impacted by the net electroneutral anion exchange pathways, so these results cannot provide functional insight into the upregulation of *CA‐*c, *nbc, slc26a3a,* and *slc26a6*.

A second fundamental component of osmoregulatory plasticity in euryhaline fishes is the ability for the gills to transition between absorptive and excretory epithelia. Our previous work on red drum demonstrated a significant downregulation in *cftr* and *nkcc1* within the first 24 h posttransfer, which generally agrees with similar work on other species (Scott et al. 2004; Bodinier et al. 2009; Kang et al. 2013). The response upon transition into seawater was much slower; however, there was a trend toward upregulation at 24 h for *cftr*. Significant upregulation of *cftr* and *nkcc1* did not occur until 72 h posttransfer, and the level of expression was similar to those found from the gills of fish never exposed to freshwater. The expression of both genes decreased at 7 days posttransfer, which highlights the potential transient responses in mRNA expression. While the relatively slow branchial osmoregulatory transition was initially surprising, these data fit well within the time course of intestinal remodeling and the onset of drinking. The available evidence suggests that transition to seawater does not cause a large effect on plasma ion chemistry, and therefore the switch to branchial ion excretion mechanisms may not be required until fish begin to absorb large concentrations of ions through esophageal desalination and further intestinal processing of imbibed fluid. The 72 h upregulation in *cftr* and *nkcc1* correlates well with the peak intestinal water volume and presumed gastrointestinal ion absorption.

### Perspectives

To our knowledge this is the first study to undertake a comprehensive analysis of both bicarbonate‐dependent and ‐independent absorption pathways in the same experimental series. Previous studies have largely focused on only a subset of these pathways and therefore have provided an incomplete representation of intestinal plasticity. An important finding from these experiments was that *nkcc2* was the most responsive of the intestinal ion transporters when transitioning from freshwater to seawater, although *CA‐c* was equally responsive. These gene expression data were verified at the functional level using short‐circuit current analysis, which clearly demonstrated an almost 50% increase in absorptive current in seawater relative to freshwater. It has long been known that *nkcc2* plays a role in ion absorption that drives water uptake in the marine fish intestine (Frizzell et al. 1979; Field et al. 1980), yet the role of this protein has been curiously overlooked in many of the studies of euryhaline fishes. The more recent work has focused mostly on the primary and ancillary components of the bicarbonate secretion pathway (Grosell et al. 2007, 2009), in part owing to the potential impacts on oceanic carbon cycling (Wilson et al. 2009). While it is clear that these two pathways likely work in concert, owing to a described feedback loop initiated by soluble adenylyl cyclase (Tresguerres et al. 2010), it is important to note that *nkcc2* likely plays a more important role than generally considered. An interesting hypothesis is that *nkcc2* may be a fast‐response pathway that is crucial in periods of osmoregulatory stress, while bicarbonate secretion is a steady‐state pathway that can maximize water uptake efficiency through CaCO_3_ precipitation. Under this scenario, the upregulation of *nkcc2* would help to explain the excellent tolerance of acute seawater transitions by red drum.

## Conflict of Interest

None declared.
